# Nonideal resistive and synaptic characteristics in Ag/ZnO/TiN device for neuromorphic system

**DOI:** 10.1038/s41598-021-96197-8

**Published:** 2021-08-16

**Authors:** Jongmin Park, Hojeong Ryu, Sungjun Kim

**Affiliations:** Division of Electronics and Electrical Engineering, Dongguk University, Seoul, 04620 South Korea

**Keywords:** Electrical and electronic engineering, Nanoscale devices

## Abstract

Ideal resistive switching in resistive random-access memory (RRAM) should be ensured for synaptic devices in neuromorphic systems. We used an Ag/ZnO/TiN RRAM structure to investigate the effects of nonideal resistive switching, such as an unstable high-resistance state (HRS), negative set (N-set), and temporal disconnection, during the set process and the conductance saturation feature for synaptic applications. The device shows an *I–V* curve based on the positive set in the bipolar resistive switching mode. In 1000 endurance tests, we investigated the changes in the HRS, which displays large fluctuations compared with the stable low-resistance state, and the negative effect on the performance of the device resulting from such an instability. The impact of the N-set, which originates from the negative voltage on the top electrode, was studied through the process of intentional N-set through the repetition of 10 ON/OFF cycles. The Ag/ZnO/TiN device showed saturation characteristics in conductance modulation according to the magnitude of the applied pulse. Therefore, potentiation or depression was performed via consecutive pulses with diverse amplitudes. We also studied the spontaneous conductance decay in the saturation feature required to emulate short-term plasticity.

## Introduction

High-performance and high-speed memory devices are required for application to new computing systems to satisfy the requirements of rapid information processing in the era of the Internet of things and big data. Existing charge-based memories, such as dynamic random-access memory and flash memory, have various disadvantages. Resistive random-access memory (RRAM), a type of conventional resistance-change-based nonvolatile memory, is preferred owing to its low power consumption, high density, and fast response speed^1–5^. Moreover, the multistate conductance, which depends on voltage pulses, facilitates the production of artificial synaptic devices in neuromorphic computing systems^6–17^.

RRAM structures using various materials have been reported. Among diverse metal oxides, amorphous metal oxide semiconductors, such as ZnO^18–24^, SnO^25^, and indium gallium zinc oxide^26^, are suitable for the development of synaptic devices because of their gradual resistance switching change. ZnO is an n-type semiconductor material with a wide bandgap (~ 3.3 eV) and exhibits electrochemical activity based on the reduction–oxidation process of resistive switching^18–24^. Various resistive switching behaviors have been discovered depending on the top and bottom electrodes and the process conditions^18–24^. For example, a Pt/ZnO/Pt device with inert Pt exhibits a resistive switching mechanism as a valence change memory activated by oxygen vacancies^21^. In contrast, an Ag/ZnO/Pt device shows a good performance in terms of endurance and retention as an electrochemical metallization memory based on the Ag cation movements^22^. However, Pt electrode is not suitable for mass production because of high price and the difficulty of etching in the standard semiconductor process. Depending on the impact of the oxygen composition of ZnO in an Al/ZnO/Al device^23^, unipolar resistive switching shows a larger ON/OFF ratio, lower switching voltages, and superior endurance at the highest O_2_/Ar gas flow rate^24^. However, the nonideal resistive switching behaviors of ZnO-based RRAM, such as N-set behavior and unstable filament formation, can have a negative effect on resistive switching^27,28^.

In this study, the use of Ag as an anode, the rates of the redox process, and ion mobility influenced the forming process of the filaments. When a voltage is applied to the anode, Ag nanoclusters are formed through an oxidation process. They drift along the electric field in the insulator to form an Ag bridge through a reduction process at the counter electrode. TiN, which is used as the cathode, acts as an oxygen reservoir when a positive bias is applied, increasing the oxygen vacancies inside the insulator. It is important to detect nonideal resistive switching before synaptic characteristics are achieved. Investigations of nonideal resistive switching behavior have seldom been reported. We investigated nonideal resistive switching in terms of the formation and dissolution of filaments. As a positive bias is applied to the Ag electrode in the switching process, Ag atoms have the greatest impact on filament growth; however, undesirable switching behaviors, such as N-set and temporal disconnection, are induced by the oxygen vacancies that occur in the reset process and increase instability in the device performance.

Moreover, it is important to detect the nonideal behaviors in gradual conductance changes when applying pulses to the Ag/ZnO/TiN device in a neuromorphic system. To prevent the N-set, the reset voltage should be as low as possible. We ensured that multilevel states emulated the synaptic weight of synaptic devices in neuromorphic systems. However, the stack of Ag/ZnO/TiN showed a rapid increase in conductance through the uncontrollable Ag nanoclusters, and these saturated conductance characteristics could be minimized by adjusting the pulse magnitude. We also observed that the conductance drift decreased, as the strength of the conducting filament increased.

## Methods

The Ag/ZnO/TiN device was prepared as follows. TiN was deposited via DC sputtering by reacting Ti and oxygen on a SiO_2_/Si wafer. A 10-nm-thick ZnO layer was deposited via pulsed DC sputtering on a TiN/SiO_2_/Si substrate. A Zn source target was used to react with oxygen for ZnO film deposition. Ar (6 sccm) and O_2_ (14 sccm) gases were passed through the chamber during deposition. The sputtering power and pressure were 0.1 kW (pulsed DC, 50 kHz) and 1 mTorr, respectively. Finally, a 100-nm-thick Ag top electrode was deposited on the ZnO layer using an e-beam evaporator. Each cell was patterned using a shadow mask with a diameter of 100 µm. As a comparative sample, Ag/ZnO/Pt device was fabricated as a comparative sample. Here, a-100 nm-thick Ag was deposited by e-beam evaporator. The electrical properties were characterized in the DC mode using a Keithley 4200-SCS semiconductor parameter analyzer in the pulse mode using a 4225-PMU ultrafast module. For all the electrical measurements of the sample, the external bias of the semiconductor measurement unit and pulse measurement unit was applied to the Ag top electrode, whereas the TiN bottom electrode was grounded.

## Results and discussion

**Figure 1:**
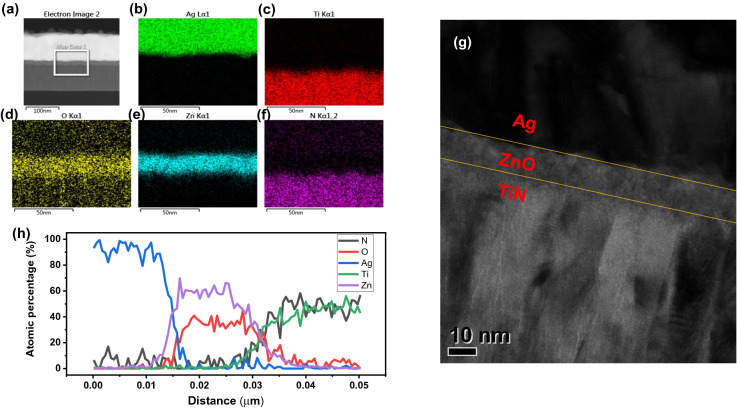
Material and chemical analyses of the Ag/ZnO/TiN device: (**a**) STEM image for EDS mapping. EDS mapping image with different elements: (**b**) Ag, (**c**) Ti, (**d**) O, (**e**) Zn, (**f**) N. (**g**) TEM image. (**h**) EDS line scan.

Figure 1a shows the scanning transmission electron microscopy (STEM) image of the Ag/ZnO/TiN device, and Fig. 1b–f show the distribution of the components (Ag, Ti, O, Zn, N) obtained through energy-dispersive X-ray spectroscopy (EDS) mapping. Each layer of the Ag/ZnO/TiN device is distinguishable. In addition, the thickness of ZnO was measured using a high-resolution transmission electron microscopy (TEM) image, as shown in Fig. 1g. Moreover, the atomic concentration of each component was scanned using an EDS line scan, as shown in Fig. 1h.

**Figure 2:**
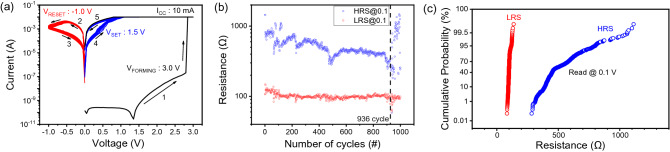
(**a**) Typical *I–V* characteristics of the Ag/ZnO/TiN device. (**b**) Endurance testing involving 1000 cycles. (**c**) Cumulative probability of HRS and LRS with a read voltage of 0.1 V.

First, the basic electrical characteristics of the Ag/ZnO/TiN device were determined using a DC dual sweep. The device requires a forming process with a forming voltage (V_forming_) of approximately 3 V. A significant increase in current was observed during the forming process with a compliance current (CC) of 10 mA, which prevented permanent breakdown during the formation of conductive filaments, as shown in Fig. 2a. Subsequently, the *I–V* curves with 30 consecutive ON/OFF cycles were conducted with set and reset occurring in the negative and positive biases in the bipolar resistive switching (BRS) mode, respectively. The current in the low-resistance state (LRS) gradually decreased when a negative voltage of approximately − 1.0 V (reset voltage, V_reset_) was applied to the top electrode (TE) after the formation of the device, and the state of the device changed from LRS to a high-resistance state (HRS). The switched HRS changed to LRS under a positive voltage sweep from 0 to 1.5 V (set voltage, V_set_). Subsequently, 1000 successive cycles of ON/OFF switching were performed to investigate the impact of instability in the HRS, as illustrated in Fig. 2b. As the read voltage of 0.1 V exhibits the largest ON/OFF ratio, it was used to verify the variation between HRS and LRS during the endurance testing. In the repetitive switching procedure, the ON/OFF ratio decreased gradually from 8 to 3 until a switching failure occurred at the 936th cycle. Although the switching failure occurred so that the conductance failed to revert to the low-conductance state, the switching behavior was observed at the 937th cycle owing to the spontaneous decay in filaments. However, the instability and fluctuations in the HRS became severe after the switching failure. The fluctuations in resistance before the switching failure are shown by the cumulative probability, as shown in Fig. 2c. The HRS varied from 283 to 1112 Ω, whereas the LRS was measured to be stable from 80 to 130 Ω.

**Figure 3:**
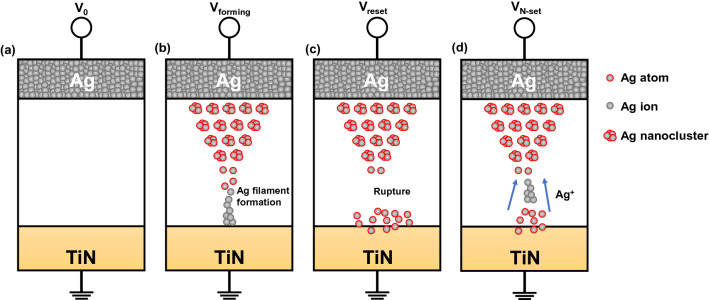
Schematic for the switching mechanism of the Ag/ZnO/TiN device. (**a**) Initial state. (**b**) Forming state. (**c**) Reset state. (**d**) Negative-set state.

Figure 3a shows a schematic of the Ag/ZnO/TiN RRAM structure in the pristine state. Ag nanoclusters are formed through oxidation when V_forming_ is applied to the device. The formed Ag clusters drift to the counter electrode via the electric field and facilitate the reduction process at the cathode, eventually forming filaments inside the insulator, as shown in Fig. 3b. In Fig. 3c, the oxidation process occurs in the Ag bridge because of the negative voltage of the reset process; consequently, a dissolution that results in a change from a high-conductance state to a low-conductance state occurs. However, the Ag filament can be reconnected with a larger voltage sweep amplitude, as shown in Fig. 3d. Ag ions remaining on the TiN side enter the ZnO again, and then the N-set occurs. A similar phenomenon and the mechanism of the N-set were revealed in Ag/ZnO/Pt devices^28^.

**Figure 4:**
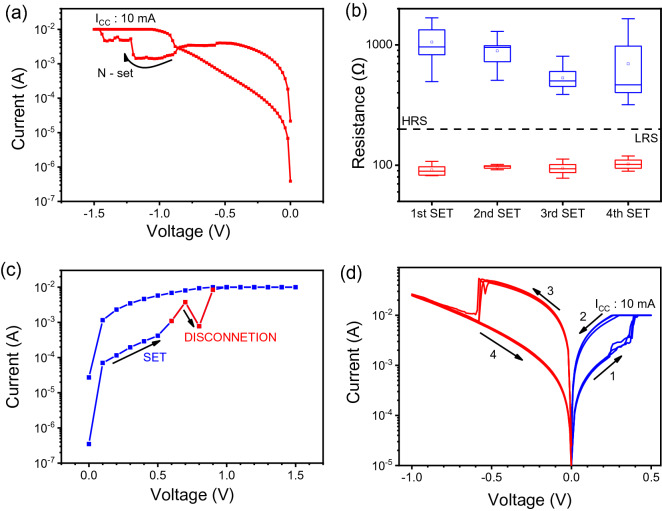
Nonideal resistive switching. (**a**) N-set behavior after reset. (**b**) HRS and LRS distributions after N-set. (**c**) Temporal disconnection of filament in the HRS. (**d**) Typical I–V characteristics of Ag/ZnO/Pt device.

Two undesirable resistive switching behaviors, namely, N-set after positive forming and temporal disconnection in resistive switching, can be observed in the *I–V* curves (Fig. 4). Under the negative voltage sweep from 0 to − 1.5 V (V_N-set_), the current gradually decreased before increasing to approximately − 1.3 V. Despite the current reaching the CC of 10 mA, the conductance was recovered to the low condition as in Fig. 4a. A CC of 10 mA was also used in the negative bias to prevent permanent breakdown as a result of the oxygen vacancies. To investigate the impact of the N-set on the performance of the device, we performed three intentional N-sets between the 10 consecutive ON/OFF cycles, as shown in Fig. S1. The average value of the HRS decreased and the fluctuations were maintained; however, in the final sequence, the opposite results were achieved. In contrast to the increase in HRS, the variations in the HRS became severe, as shown in Fig. 4b. The N-set, as well as the temporal disconnection shown in Fig. 4c, can be one of the reasons for the unstable HRS. The instability is triggered by the dissolution of the filaments and occurs at a positive voltage of approximately 0.8 V. A more plausible explanation is presented in Fig. S2. To clearly observe the effect of N-set in RRAM device, we prepared a comparative device. It is noted that Pt bottom electrode is used to minimize the oxygen accumulation effect between ZnO and bottom electrode. Pt could minimize the effect of oxygen vacancies between the ZnO and bottom electrode due to its inert characteristic. Figure 4d shows the I–V curves of Ag/ZnO/Pt device. In comparison with the Ag/ZnO/TiN device, Ag/ZnO/Pt device does not show the N-set or temporal disconnection during the switching operation. From these observations, the recombination of oxygen atoms caused by the oxygen reservoir ability of TiN could impair the stability of the resistive switching in Ag/ZnO/TiN device.

**Figure 5:**
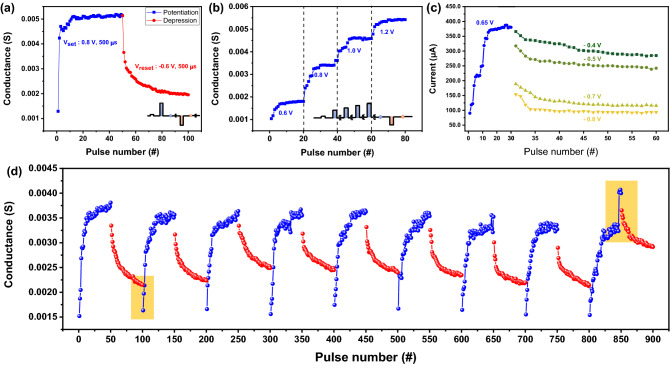
Synaptic behaviors of the Ag/ZnO/TiN device. (**a**) Potentiation and depression characteristics with a set pulse (0.8 V, 500 μs) and a reset pulse (− 0.6 V, 500 μs). (**b**) Gradual stepwise increase in conductance via an incremental pulse scheme. (**c**) Conductance modulation by varying the reset pulse voltage. (**d**) Multiple potentiation and depression curves.

Variable conductance is essential for mimicking a synaptic network. We demonstrate the modulation of the conductance in the Ag/ZnO/TiN device with pulses of various magnitudes and widths. Figure 5a displays the potentiation or depression that was modified using identical pulses. The Ag/ZnO/TiN device exhibits a synaptic response of saturated conductance similar to other conventional RRAM devices, which show a nonlinear synaptic response in the form of potentiation or depression. The identical pulses include positive or negative pulses for potentiation and depression, with the amplitudes of 0.8 and − 0.6 V, respectively. All the pulse widths are fixed at 500 μs and the interval between the pulses is 1 ms. Although the saturation feature is shown in the conductance modulation, it is possible to mimic a synaptic device that shows a better linear synaptic response using the nonidentical pulses.

When applying nonidentical pulses whose amplitude increases after every 20 pulses in steps of 0.2 V from 0.6 to 1.2 V, the synaptic weight in the potentiation can be modulated into a stair format as shown in Fig. 5b. Similarly, the depression shows saturation features with the magnitude of the pulses, as shown in Fig. 5c. However, the shape of the conductance is not fixed and becomes unstable when the nonidentical pulses are applied 15 times, as shown in Fig. S4. Accordingly, although the synaptic weight can be increased by adjusting the magnitude of the pulses, it is difficult to maintain a constant conductance owing to the collapse in the format of conductance modulation. Figure 5d shows nine repeated potentiation and depression cycles in one cell, where the set pulse conditions are 0.8 V and 300 μs and the reset pulse conditions are − 0.6 V and 500 μs. After the completion of one potentiation/depression cycle, the next cycle was performed. Overall, the nine cycles showed a similar trend; however, in the ninth cycle, a nonideal occurrence of a sharp increase in conductance at the 48th point of potentiation was observed. Note that the conductance of the first point of a cycle decreased more than the conductance of the last point of the previous cycle. This can be explained by the short-term effect, where the conductance of the device is somewhat lowered if it is not fully reset to the minimum point at which it can move.

**Figure 6:**
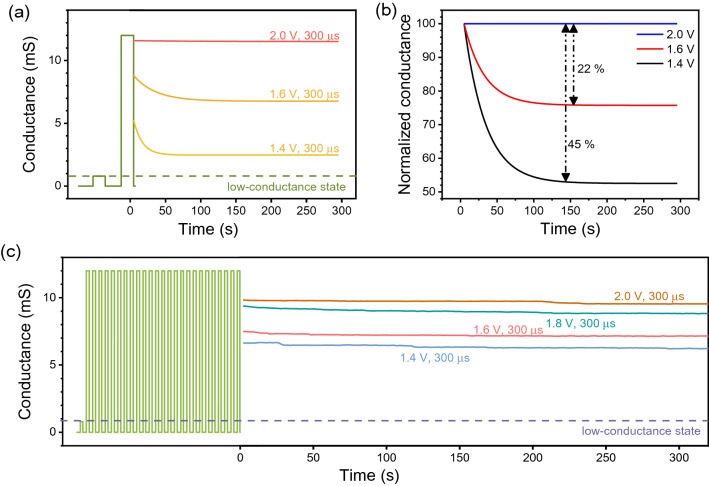
STP and LTP characteristics of the Ag/ZnO/TiN device. (**a**) Current decay characteristics after one pulse response. (**b**) Normalized conductance decay for the observation of STP. (**c**) LTP by multiple pulse inputs with different pulse voltage conditions.

Subsequently, the short-term effect was quantitatively investigated by varying the pulse conditions. The plasticity of the synaptic device is also necessary to mimic the learning properties of biological synapses. The short-term plasticity (STP) and long-term plasticity (LTP) can be observed after the pulse response, as shown in Fig. 6. Here, the focus was made on the time scale of the part to which the pulses were applied. The conductance levels, measured using a voltage of 0.1 V at every 5 s, differed by the magnitude of the given pulse and varied from a low-conductance state of 1 ms to a high-conductance state of 12 ms in Fig. 6. Through the normalized conductance, it is possible to check the amount of decrease compared with the initial value, as shown in Fig. 6b. In the cases where the pulses of 1.4 V and 1.6 V were applied for 150 s, the decreases were observed to be approximately 45% and 22%, respectively. Although the conductance was increased by only one pulse with apparent STP characteristics, the device failed to recover the HRS spontaneously, as shown in Fig. 6a. As the Ag bridge inside the insulator was formed by the metal nanoclusters, the imperfect nanoclusters decayed over time, whereas the stabilized Ag clusters remained in the filaments. The remaining clusters prevented the filaments from being completely dissolved, and filament decay did not occur perfectly. Hence, the spontaneous decay in the Ag bridge became smaller with an increase in the amplitude of the pulses applied to the device, as it stabilized the imperfect Ag clusters inside the filaments. In Fig. 6c, 25 pulses were applied, and the current decay was monitored. Here, except for the number of pulses applied, the same pulse conditions as in Fig. 6a were applied. The device displayed LTP with a very small percentage of decay. Although the LTP feature could be achieved by applying strong stimulation, the STP showed an unsteady performance to emulate the synaptic response owing to the saturation feature.

## Conclusion

In this study, we investigated the nonideal resistive switching and synaptic characteristics of Ag/ZnO/TiN devices. First, the thickness and material components of the device stack were investigated via TEM and EDS analyses. The BRS with gradual switching was achieved after the electroforming process. The N-set occurred at a large negative sweep voltage, which could cause device failure. An unstable HRS was observed before the set process, which could adversely affect the gradual change in resistance. Second, multiple conductance states were achieved with different pulse conditions. Nonideal cases, such as conductance-saturated behavior and current decay with time, were investigated. Finally, we demonstrated both LTP and STP, which were obtained by controlling the strength of the pulse input.

## Supplementary Information

Supplementary Information 1.
