# Supplementary material for: Nonideal resistive and synaptic characteristics in Ag/ZnO/TiN device for neuromorphic system
Source: Sci Rep. 2021 Aug 16;11:16601. doi: 10.1038/s41598-021-96197-8 (PMC8367949; doi:10.1038/s41598-021-96197-8)
Supplement: Supplementary file 1 — Supplementary Information 1. [file 41598_2021_96197_MOESM1_ESM.docx]

**Nonideal resistive and synaptic characteristics in Ag/ZnO/TiN device for neuromorphic system**

Jongmin Park, Hojeong Ryu, Sungjun Kim^∗^

Division of Electronics and Electrical Engineering, Dongguk University, Seoul 04620, South Korea

Correspondence: S. Kim ([sungjun@dongguk.edu](mailto:sungjun@dongguk.edu))

Fig. S1 Intentional N-set behavior with the repetitive 10 ON/OFF cycles.

000

Fig. S2 (a) I-V curves for temporal disconnection of filament. (b) Schottky emission fitting at below 0.6 V. (c) Schottky emission fitting at higher than 0.8 V. A positive reset in Fig. S2(a) is induced for several times under the negative forming. Compared with those two curves in a positive bias, the HRS follows Schottky emission below the voltage of 0.6 V and the current decreases from 0.6 V to 0.8 V. Despite the decrease in current, the conduction mechanism follows Schottky emission again when the voltage of more than 0.8 V is applied.

Fig. S3 Worsened instability by the 15 successive conductance modulations.

Fig. S4 Worsened instability by the 15 successive conductance modulations (arranged with the linearity curve).
